# High-Precision Ultra-Long Air Slit Fabrication Based on MEMS Technology for Imaging Spectrometers

**DOI:** 10.3390/mi14122198

**Published:** 2023-11-30

**Authors:** Xiaoyu Ren, Selina X. Yao, Jiacheng Zhu, Zejun Deng, Yijia Wang, Baoshun Zhang, Zhongming Zeng, Hao Zhai

**Affiliations:** 1 Nanofabrication Facility, Suzhou Institute of Nano-Tech and Nano-Bionics, Chinese Academy of Sciences, Suzhou 215123, China; bszhang2006@sinano.ac.cn (B.Z.); zmzeng2012@sinano.ac.cn (Z.Z.); 2 Key Lab of Nanodevices and Applications, Suzhou Institute of Nano-Tech and Nano-Bionics, Chinese Academy of Sciences, Suzhou 215123, China; xyren2014@sinano.ac.cn; 3 Department of Mechanical Engineering, University of Vermont, Burlington, VT 05405, USA; xiangxiaoyao@outlook.com; 4 School of Optoelectronic Science and Engineering, Soochow University, Suzhou 215006, China; zjc@suda.edu.cn; 5 School of Materials Science & Engineering, Central South University, Changsha 410083, China; zejun.deng@csu.edu.cn; 6 Institute for Advanced Study, Central South University, Changsha 410083, China; wangyijia0302@csu.edu.cn

**Keywords:** MEMS, DRIE, air slit, spectrometer

## Abstract

The increasing demand for accurate imaging spectral information in remote sensing detection has driven the development of hyperspectral remote sensing instruments towards a larger view field and higher resolution. As the core component of the spectrometer slit, the designed length reaches tens of millimeters while the precision maintained within the μm level. Such precision requirements pose challenges to traditional machining and laser processing. In this paper, a high-precision air slit was created with a large aspect ratio through MEMS technology on SOI silicon wafers. In particular, a MEMS slit was prepared with a width of 15 μm and an aspect ratio exceeding 4000:1, and a spectral spectroscopy system was created and tested with a Hg-Cd light source. As a result, the spectral spectrum was linear within the visible range, and a spectral resolution of less than 1 nm was obtained. The standard deviation of resolution is only one-fourth of that is seen in machined slits across various view fields. This research provided a reliable and novel manufacturing technique for high-precision air slits, offering technical assistance in developing high-resolution wide-coverage imaging spectrometers.

## 1. Introduction

An imaging spectrometer can simultaneously gather spatial and spectral information about a target area, collecting a data cube that can be unraveled in the spectral dimension. Thus, it enables the collection of spectral data for each point on the image, as well as collecting the image information of any spectral band. In recent decades, with rapid development speed, a series of imaging spectrometers have been developed and applied in the field of hyperspectral remote sensing aboard spacecraft [1,2,3,4,5,6,7,8]. The advancement of hyperspectral remote sensing has enabled the detection of many objects previously undetectable using broadband technology. The utilization of imaging spectrometers mounted on satellites enables various applications, such as ocean exploration [9,10], environmental monitoring, surface exploration [11], natural resources exploration [12], and biological activity monitoring [13,14,15].

The imaging spectrometer stands out due to its high spatial and spectral resolution. The growing need for accurate and efficient collection of spectral imaging data across diverse fields has prompted advancements in hyperspectral remote sensing technology, aiming for faster responses, wider coverage [16], higher spatial resolution, higher temporal resolution, and a higher signal-to-noise ratio. On the one hand, the satellite-borne spectrometer is set in a geostationary orbit, with its position relative to the ground being maintained at static and high orbit altitude. Compared to the near-earth orbit observation, it brings advantages with a higher temporal resolution and wider coverage. Moreover, improvement of the size of the view field, spatial resolution, spectral resolution, and signal-to-noise ratio, is enabled from the advance of the spectrometer [17]. Typically, an imaging spectrometer consists of an incident slit, a collimation system, a beam splitting system, and a CCD array, where the slit is located in the focal plane of the front objective of the spectrometer and the object plane of the spectrometer, which is the view field diaphragm in the spectrometer. The front objective lens captures the target images onto the slit, with the light passing through the slit into the spectral spectroscopic imaging, so the slit images of different wavelengths are obtained on the image plane, which is captured and recorded by the CCD array.

In terms of the composition and working principle of the imaging spectrometer, the performance of imaging spectrometer highly relies on the properties of the slit. In particular, the slit length determines the size of the view field of the spectrometer, while the slit width affects the spectral resolution, scanning mode, spatial resolution, and luminous flux of the spectrometer, and the uniformity of the slit width affects the uniformity of the spectral-spatial response. As spectrometers evolve into wide coverage and high resolution, the slit length becomes longer and the slit width becomes narrower. Since the geostationary orbit imaging spectrometer is not subject to the movement of the satellite platform, the exposure time can be extended to obtain enough luminous flux to achieve sufficient sensitivity, based on which the slit width can be designed to be smaller. The slit width matches the pixel size of the detector located in the focal plane, which can be narrowed to several to dozens of microns. To ensure that the spectral resolution of the slit width deviation is less than 1–2 μm, with the slit length up to several to dozens of microns, resulting in the aspect ratio exceeding more than 3 orders of magnitude, which poses a great challenge for the slit manufacturing process. Therefore, as processing accuracy of the slit becomes critical, it can significantly impact performance of imaging spectrometers.

At present, common methods of slit processing in imaging spectrometers are mainly traditional mechanical processing and laser processing. Mechanical processing is easy for volume production, but hardly it could achieve micron-level slit high-precision processing, and the slit made from sheet metal must be assembled with a fixture jig, thus the increased volume and weight makes mechanical processing difficult to be applied to the miniaturized star-bearing spectrometer [18]. Scribing and cutting techniques are typically used to fabricate the slit during mechanical processing. Due to the shear force and localized high temperatures in this process, micro-area plastic deformation occurs unexpectedly. Consequently, it could hardly achieve micron-level slit high-precision processing. The laser processing method uses high energy, the slit is cut by a small spot laser beam through the localized high-temperature ablation. The fact that it is easy for ablation to occur near the slit edge, poses a serious impact on the quality of slit defects. In contrast, the monocrystalline silicon slit utilizing the micro-electro-mechanical systems (MEMS) processing technology can be shaped simultaneously through reactive ion etching (RIE) without causing any mechanical damage or internal stress near the material surface. The graphical accuracy of the slit depends on the photolithography machine’s resolution, which is generally lower than a hundred nanometers. Moreover, multiple slit units can be manufactured on a single wafer using the same process conditions. Thus, the MEMS technology can be used for the fabrication of silicon-based high-aspect-ratio slit, it provides higher precision, uniformity, and controlled defects, readily achievable for mass production [19]. However, the MEMS slit has limitations on shock and vibration resistance, which is attributed to its thin edge thickness, with only a few tens of microns, and its lower than metal mechanical strength. Typically, the MEMS technology is applied in the manufacturing process of semiconductor chips at a scale ranging from microns to nanometers. The dimensions of the resulting device unit are of a similar magnitude in both length and width, with the units arranged in an array on a wafer, numbering over a thousand. The chip with defects caused by random particles can be identified and removed during later sorting. As the length-to-width ratio of the slit spans over three orders of magnitude, defects on the slit can magnify the impact exponentially. This poses new challenges in the manufacturing of high-precision, low-defect ultra-long slits using MEMS technology. Furthermore, when the thickness of single crystal silicon material is in the order of tens of microns, its visible wavelengths exhibit some degree of transmittance. Therefore, a high-quality coating is needed on the surface to suppress the interference of stray light on the spectral imaging.

## 2. Materials and Methods

**Figure 1:**
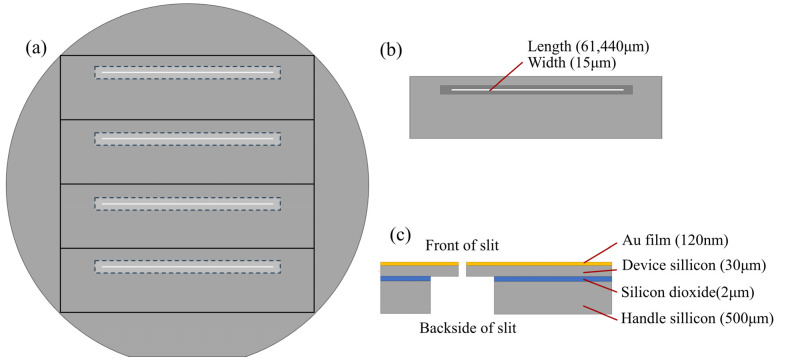
(**a**) Front side of 4-inch slit wafer. (**b**) Back side of the slit. (**c**) Cross-section of the slit.

The air slit structure designed in this study was shown in Figure 1, which was fabricated on a 4-inch SOI wafer using MEMS manufacturing processes. The length of the slit is designed based on the Geostationary Orbit Full Spectrum Wide Coverage High Fidelity Imaging Spectrometer (GeoFWHIS) [20], which has 16,000 spatial sampling points in the VNIR band and an instantaneous view field of up to 0.7 μrad. The characteristics of wide coverage and high spatial resolution also demanded a long slit in the system, which can be reduced by splicing multiple spectroscopic imaging systems, however, the length of the designed slit was still more than 60 mm. Meanwhile, the width of the slit was matched to the size of the detector element at the focal plane, with a design width of 15 μm, an accuracy of ±10%, and a slit edge thickness of 30 μm. The SOI wafer had a thickness of 500 μm, <100> orientation, a diameter of 4 inches, a top silicon layer thickness of 30 μm, and a buried silicon dioxide layer thickness of 2 μm.

**Figure 2:**
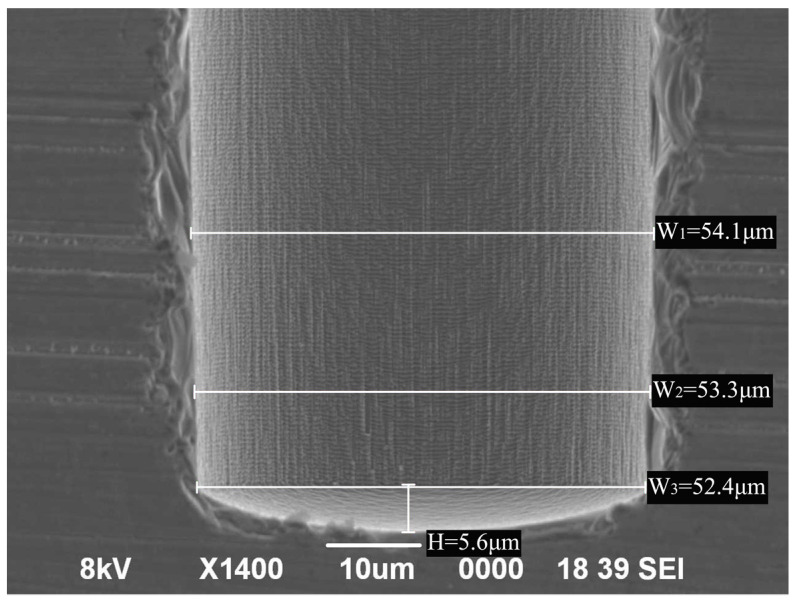
DRIE BOSCH process etched bottom and sidewalls in SEM.

The air slit comprises a metal layer, a device silicon layer, a silicon dioxide buried layer, and a handle silicon layer. Each spectral line generated by the dispersive spectroscopy of the spectrometer serves as an image of the slit, as the optical signal passes through the slit. Therefore, to ensure uniform resolution of spectral lines within the view field, it is imperative to have consistent slit width, burr-free edges, perpendicular and straight sidewalls, and smooth surfaces. In this paper, we used a Deep Reactive Ion Etching (DRIE) process called the “BOSCH Process” to ensure the perpendicularity and straightness of the slit sidewalls. The Bosch process is a repeating sequence of etching and passivation processes, which is a vertical and anisotropic etching technique invented by Robert Bosch GmBH [21]. However, during the etching process, the bottom becomes irregular [22], as depicted in Figure 2, resulting in a non-uniform slit edge after etching through. As DRIE cannot etch SiO_2_, a buried oxide layer was used in the SOI sheet as the cutoff layer for DRIE etching [23], and the width of the bottom of the slit was controlled precisely by controlling the process parameters and over-etching both sides of the SOI sheet to ensure the edge quality. After completing the slit etching process, the SiO_2_ at the bottom of the slit was removed through wet etching.

**Figure 3:**
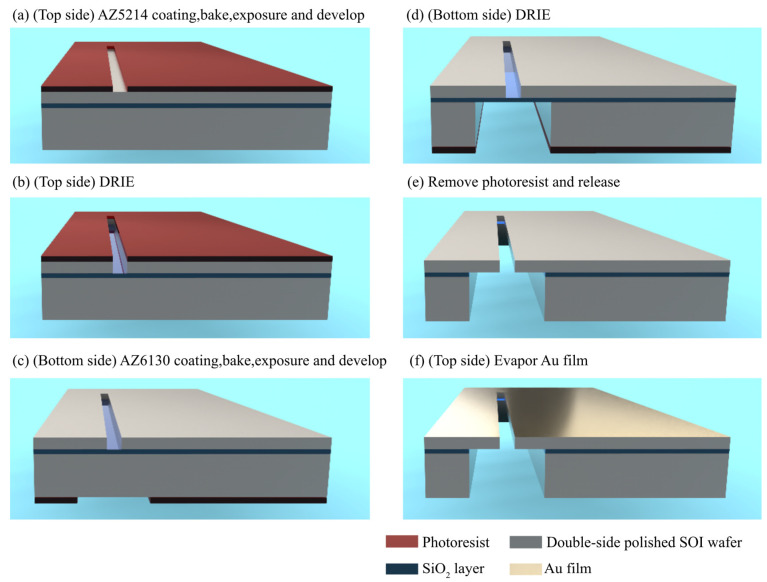
The overall fabrication process of the proposed MEMS air slit.

The silicon handle within the slit structure, measuring 500 μm in thickness, provided crucial mechanical support to the silicon slit edge. The edge was incredibly thin, measuring only 30 μm and exhibiting a remarkable length-to-thickness ratio exceeding 2000:1. An electron beam evaporation device evaporated a metal layer across the front of the slit unit, effectively blocking the transmission of light through anything other than the slit itself, i.e., neither from the slit edge nor any other part of the slit. The overall fabrication process of the proposed MEMS air slit was illustrated in Figure 3.

The proposed MEMS air slit in this study was fabricated using a double-sided polished 4-inch SOI wafers, underwent ultrasonic cleaning using acetone, IPA and polydimethylsiloxane pretreatment to enhance the adhesion of the photoresist coating on the sample surface. The topside was spin-coated with AZ-5214 photoresist at 4000 rpm and baked. Figure 3a displayed that the top side underwent exposure with a broadband (365–425 nm) UV light source (MA6 Mask Aligner, SUSS Co., Ltd., Garching, Germany) using the Photomask-A with the slit pattern. After developing and curing, the top side was subjected to etching using BOSCH process, as demonstrated in Figure 3b. The etching rate and time were adjusted to achieve slight over-etching and ensure the removal of bottom silicon on the silicon oxide layer. Subsequently, the original photoresist was removed before spin-coating the bottom side of the wafer with AZ-6130 photoresist. After baking, sample markers were aligned with the markers of photomask-B, which had a slit backside pattern, using a backside microscope of the MA6, as depicted in Figure 3c. Upon the completion of developing and curing, the bottom side was subjected to etching using BOSCH process, as demonstrated in Figure 3d. he etching rate and time were adjusted to achieve slight over-etching and ensure the removal of bottom silicon on the other side of the silicon dioxide layer. The silicon dioxide layer was removed with BOE solution to release the air slit, as demonstrated in Figure 3e. An electron beam evaporation equipment (EI-5Z, ULVAC Co., Ltd., Chigasaki, Japan) was utilized to deposit a 120 nm layer of metallic Au on the front side of the slit, as shown in Figure 3f.

**Figure 4:**
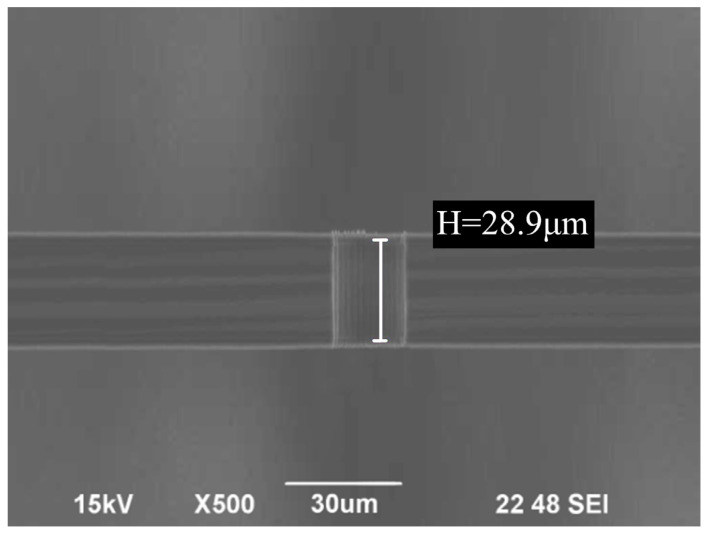
SEM image of slit cross-section.

As shown in Figure 4, the slit cross-section indicated that the sidewalls were perpendicular and straight, with noticeable microstructural undulations on their surface. As the BOSCH process alternated between flowing passivation gas C_4_F_8_ and etching gas SF_6_ in the reaction chamber [24], a transitional process occurred between the deposition and etching in each repeated cycle, which is attributed to the periodic scallop structures on the slit sidewalls [25]. As a result, the roughness of the slit sidewalls was affected. As an approach to reduce the undulation height of the sidewall and alter the scallop pattern cycle, while also minimizing the roughness of the slit sidewall, the ratio of etching and passivation during the etching cycle was adjusted and the amount of Platen HF during etching was reduced.

In this paper, the three-step BOSCH process was used [24]. Table 1 showed the average values of cycle and amplitude of the scallop structures with varying etch parameters. Recipe 2 maintained the same passivation to etch cycle time and ratio as recipe 1 and reduced the platen HF during Etch1 by about 20%. Meanwhile, Recipe 3 reduced the passivation time by 27% and the Etch2 time by 33%, while maintaining the Etch1 platen HF constant.

**Figure 5:**
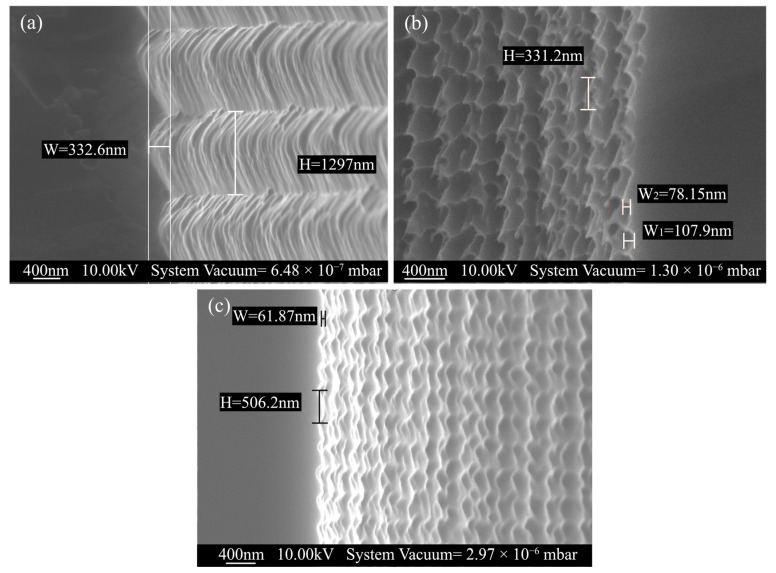
Cross-sectional SEM image of the fabricated DRIE scallop. (**a**) Recipe 1. (**b**) Recipe 2. (**c**) Recipe 3.

SEM images of the etched cross sections of the three recipes were presented in Figure 5. Recipe 1 demonstrated a mean scallop structure amplitude value of 332.6 nm, while Recipe 2 exhibited a mean value of 93.0 nm. The roughness was greatly enhanced by Recipe 2, however, the scallop morphology displayed a distinct angular bulge. Recipe 3 exhibited the lowest roughness in the scallop structure, and its overall morphology appeared to be straighter and smoother.

## 3. Results and Discussion

**Figure 6:**
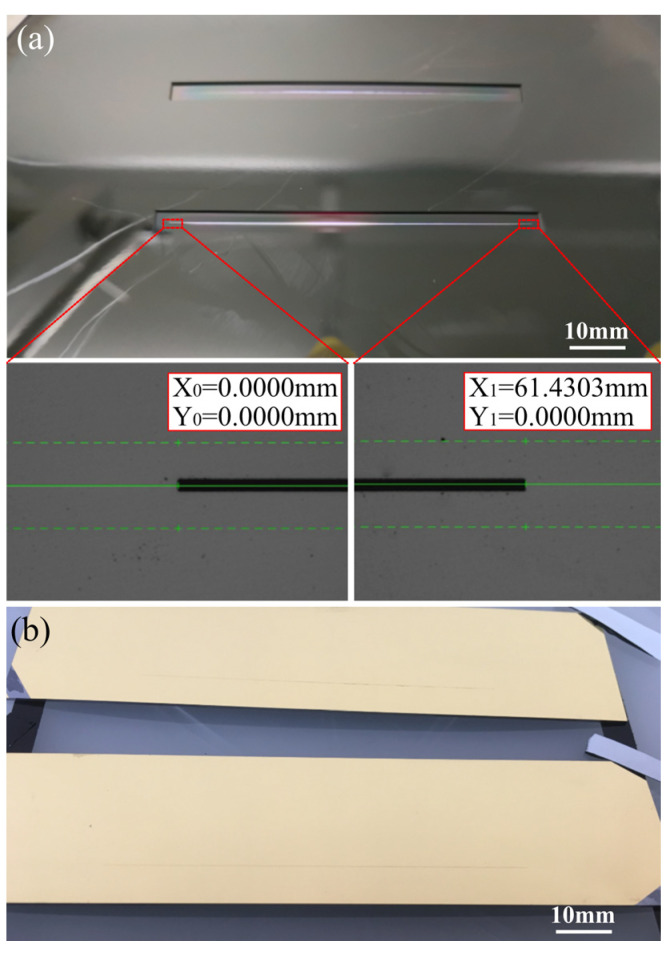
(**a**) Backside of MEMS air slit wafer. (**b**) Front side of fabricated MEMS air slit after dicing.

In this paper, the silicon substrate’s long slit generated by the MEMS technology was designed to surpass 60 mm in length. Precise measurement of this dimension wasn’t feasible using an ordinary optical microscope. A Wafer Dicing Saws (DFD641, DISCO Co., Ltd., Tokyo, Japan) was utilized to level the slit and measure its length, yielding a value of 61.43 mm, as depicted in Figure 6.

**Figure 7:**
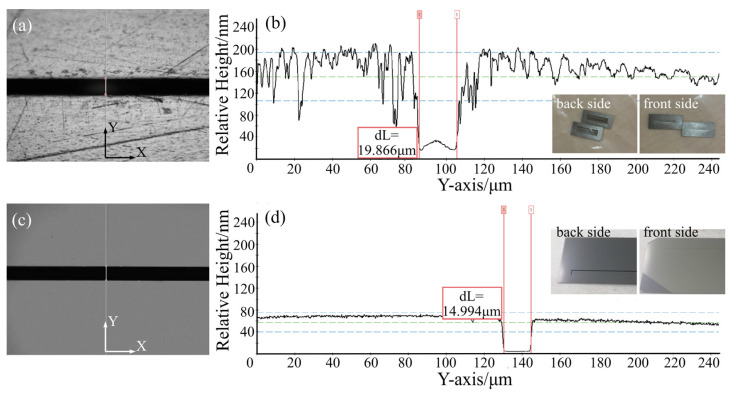
(**a**) Micrograph of machined air slit. (**b**) Machined slit profiler test curves. (**c**) Micrograph of MEMS air slit. (**d**) MEMS slit profiler test curves.

Typically, it takes a few steps in the fabrication of machined slits. Firstly, the slit sidewalls are scribed and cut with a maximum machining accuracy of ±3 μm. Secondly, the slit edge area is grinded with grinding wheels, reducing the average edge thickness to 30 μm. Finally, two edges are joined together to form a complete slit with a splice accuracy of ±2 μm. The mechanical slits in this paper were screened samples with the highest processing and splicing accuracy. As depicted in Figure 7a,c, the MEMS slit and machined metal slit were observed and compared under a 10× optical microscope. The edge of the MEMS slit appeared straighter and sharper compared to the machined slit, which exhibited clear curvature at its edge. As per the measurement of the profilometer tests, shown in Figure 7b,d, the roughness of the edge surface situated within 120 μm on either side of the MEMS slit was significantly less in comparison to that of the machined slit. Additionally, the measured value for the width (dL) of the MEMS slit was 14.994 μm.

**Figure 8:**
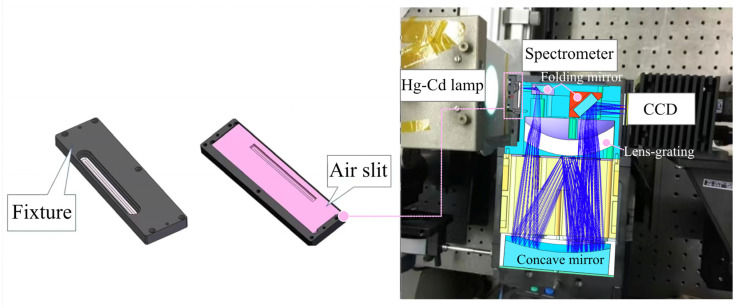
Test devices for characterizing the slit properties.

The performance test setup for the slit was illustrated in Figure 8. The spectrometer utilized the Wynne-Offner configuration with a blazed grating for superior imaging performance. It had superior anastigmatism performance and allowed a longer slit while maintaining compactness. Meanwhile, it maintained concentricity and inherited the distortion-free characteristic of the classical Offner configuration [26]. The system included two folding mirrors, a lens-grating and a concave mirror. The slit was mounted on the focal plane of the front objective of the spectrometer and the object plane of the spectrometer, uniformly illuminated by a Hg-Cd light source, and a CCD detector with a pixel size of 5.5 × 5.5 μm was used to capture the characteristic spectral lines of the light source. The spectral lines of Hg-Cd light sources in the visible band were distributed at 404.7 nm, 435.8 nm, 467.8 nm, 479.9 nm, 508.5 nm, 546.1 nm, 576.96 nm, 579.07 nm, and 643.8 nm. The spectral lines were sampled to characterize the visible band imaging performance of the slit.

**Figure 9:**
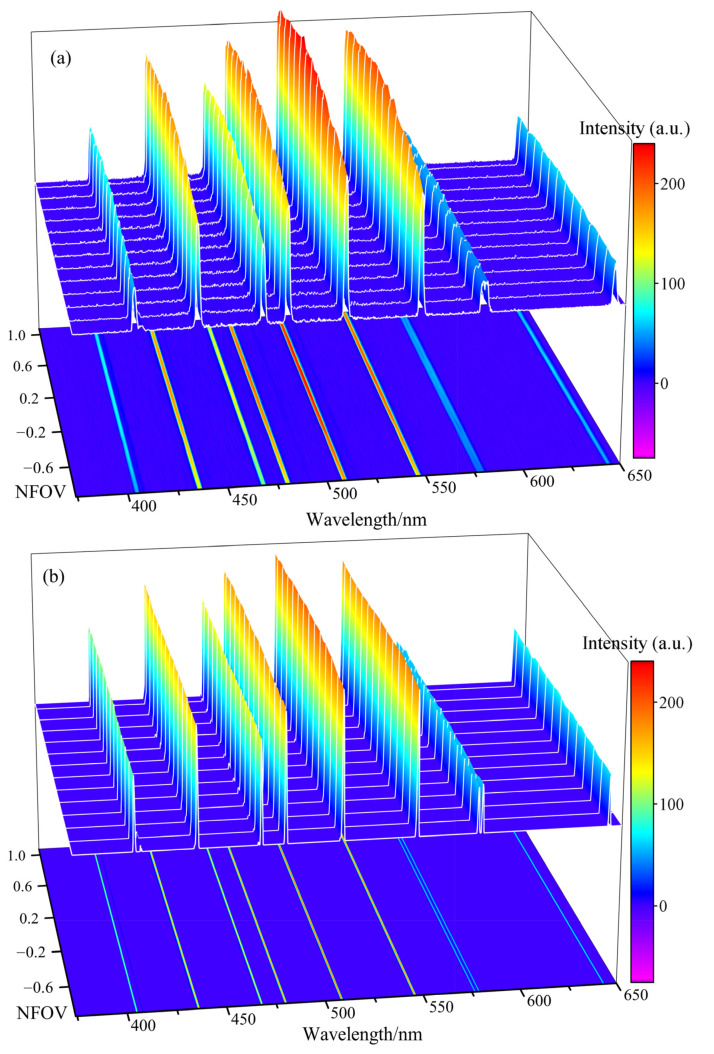
(**a**) Spectral lines intensity distribution of machined slit. (**b**) Spectral lines intensity distribution of MEMS slit.

Spectral data from different Normalized Field of View (NFOV), selected at equal spacing along the length of the spectral lines, were plotted in Figure 9. The spectral lines of the MEMS slit shown in Figure 9b, exhibited sharpness and minimal noise across various view fields and wavelengths, while the machined slit shown in Figure 9a demonstrated an average spectral noise intensity exceeding 1% of the spectral signal intensity. This is because the sidewalls of the machined slit were mechanically scribed metal surfaces that scatter stray light and induce spectral noise, while the sidewalls of the MEMS slit were scalloped microstructures etched by DRIE and covered with a polymer layer, which only reflected diffusely and did cause spectral noise. Additionally, the MEMS slit exhibited a clean spectrum without any stray light noise across the NFOV-wavelength plane, which indicated that the design and process control of the electron beam evaporation coating in the thin region of the slit edge was successful in suppressing light transmission at the slit edge while ensuring that no metal film was deposited on the slit sidewalls. The MEMS spectra of the slit exhibited consistent peak intensity of spectral lines across different view fields along the same line. Conversely, the machined slit displayed significant variations.

**Figure 10:**
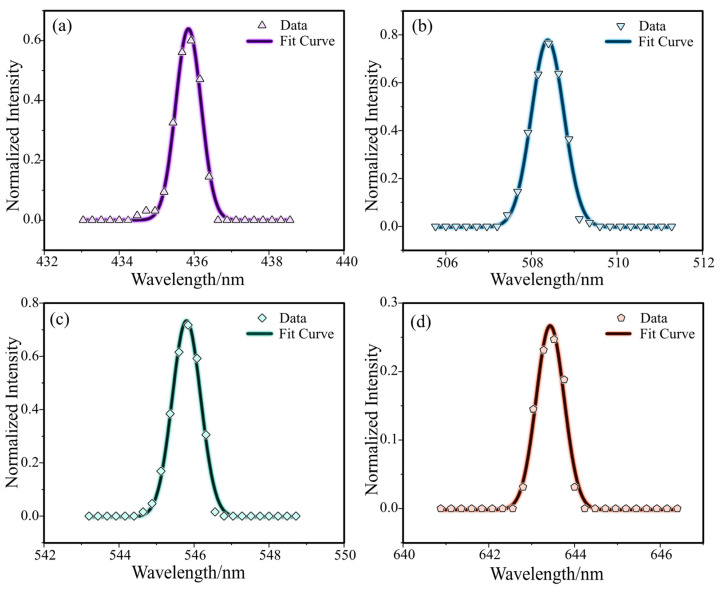
Gaussian fitting of spectral SRF curves. (**a**) 435.8 nm. (**b**) 508.5 nm. (**c**) 546.1 nm. (**d**) 643.8 nm.

To quantitatively characterize the imaging performance of the slit, the full width at half maximum (FWHM) of the spectral response function (SRF), also referred to as the spectral resolution, was typically used. Four spectral lines were selected at 435.8 nm, 508.5 nm, 546.1 nm, and 643.8 nm for characterization, the wavelengths of the four spectral lines were more uniformly distributed in the range of the visible wavelength band, and the normalized intensity of the light was distributed from low to high (0.3–0.8). The energy distribution perpendicular to the direction of the spectral lines was fitted with a Gaussian line, and the results of MEMS slit near the center view field were shown in Figure 10. The results demonstrated an excellent fit of the spectral response distribution with the Gaussian line shape, and the resolution of the four spectral lines of the MEMS slit in this field of view were obtained by calculating the FWHM as follows: 0.87 nm, 0.93 nm, 0.93 nm and 0.88 nm, respectively.

**Figure 11:**
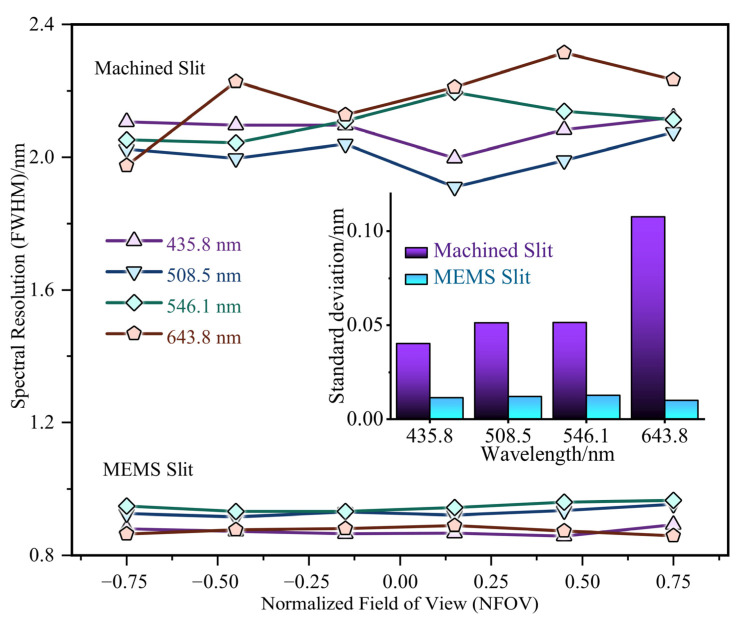
Spectral resolution (FWHM) distribution through NFOV in 435.8 nm, 508.5 nm, 546.1 nm and 643.8 nm.

A consistent methodology was used to determine the resolution at −0.75 NFOV, −0.45 NFOV, −0.15 NFOV, 0.15 NFOV, 0.45 NFOV, and 0.75 NFOV for both the MEMS and machined slit at the wavelengths of 435.8 nm, 508.5 nm, 546.1 nm, and 643.8 nm. Figure 11 listed the spectral resolution values of machined slits and MEMS slits at different wavelengths and view fields, and the MEMS slit exhibited a spectral resolution of under 1 nm in the wavelength-NFOV plane, with high stability observed across varying wavelengths, light intensities, and view fields. In contrast, the machined slit had limitations in the machining process and the metal material itself, making it difficult to achieve the accuracy of the MEMS process in terms of slit straightness and defect control. Thus, the spectral resolution distribution of the machined slit had a wider range of random fluctuations. The statistics from Figure 11 listed that the distribution of spectral resolution of MEMS slits at different view fields was more stable, with the standard deviation of spectral line resolution smaller than 0.013, which was only 1/4 of that of the machined slits.

## 4. Conclusions

In this paper, we presented a fabrication method of high-precision ultra-long slits for imaging spectrometers. To meet the demands of high-fidelity and wide-coverage imaging spectrometers, a series of methods were designed to fabricate high-performance silicon-based slits on 6-inch SOI wafers using MEMS technology. To achieve precise control of both slit width and edge quality, a high-precision etching process using silicon dioxide as the cutoff layer was developed. By adjusting the ratio of etching and passivation time during the etching cycle of the BOSCH process, the undulation of the scallop structure on slit sidewalls was reduced to 61.9 nm. To decrease the transmission of silicon slit edges in the visible wavelength range, a high-caliber electron-beam evaporation coating methodology was utilized. The high-precision MEMS slit with a length of 61.43 mm and an aspect ratio exceeding 4000:1 was successfully fabricated. The spectrometer testing bench was built and tested using a Hg-Cd light source and a Wynne-Offner system to evaluate the fabricated MEMS slit and machined slit. The spectral lines of MEMS slit were flat and straight in the visible wavelength band, with high signal-to-noise ratio, spectral line resolution < 1 nm. Moreover, spectral lines from MEMS slit were uniformly distributed in different view fields, and the standard deviation of spectral line resolution < 0.013, which was only 1/4 of that of the machined slit. As a result, the spectral imaging performance of MEMS slit was much improved compared to the mechanical slit. This paper provided a reliable new fabrication method for high-precision ultra-long air slit, which supported the development of a large-field-of-view, high-resolution imaging spectrometer.

## Figures and Tables

**Table 1:** BOSCH process condition recipes for different vertical etching depths.

Heading	Cycle	Time (s)	Platen HF	Gas(sccm)			ETCH H Each Cycle (nm)	ETCH W Each Cycle (nm)
				C_4_F_8_	SF_6_	O_2_		
Recipe1 (base)	Depo	1.1	0	370	0	0	1297	332.6
	Etch1	1.0	145	0	200	100		
	Etch2	1.2	36	0	450	0		
Recipe2	Depo	1.1	0	370	0	0	331.2	93.0
	Etch1	1.0	100	0	200	100		
	Etch2	1.2	36	0	450	0		
Recipe3	Depo	0.8	0	370	0	0	506.2	61.87
	Etch1	1.0	145	0	200	100		
	Etch2	0.8	36	0	450	0		

## Data Availability

The data that support the findings of this study are available from the corresponding author upon reasonable request.
